# The Role of Environmental Conditions on Master Marathon Running Performance in 1,280,557 Finishers the ‘New York City Marathon’ From 1970 to 2019

**DOI:** 10.3389/fphys.2021.665761

**Published:** 2021-05-17

**Authors:** Beat Knechtle, Carlyn McGrath, Olivia Goncerz, Elias Villiger, Pantelis Theodoros Nikolaidis, Thimo Marcin, Caio Victor Sousa

**Affiliations:** ^1^Institute of Primary Care, University of Zurich, Zurich, Switzerland; ^2^Medbase St. Gallen Am Vadianplatz, St. Gallen, Switzerland; ^3^Bouve College of Health Sciences, Northeastern University, Boston, MA, United States; ^4^Exercise Physiology Laboratory, Nikaia, Greece; ^5^Research Department, Berner Reha Zentrum, Bern, Switzerland

**Keywords:** running, elderly athlete, performance, environmental conditions, weather

## Abstract

**Aim:** This study investigated the influence of weather conditions on running performance in female and male age group runners in the largest marathon in the world, the “New York City Marathon.”

**Methods:** The analysis included data from 1,280,557 finishers the “New York City Marathon” from the years 1970 to 2019. Linear mixed models for men and women finishers with race time (min) as dependent variable and 5-year age groups, temperature, wind and relative humidity tertiles (low, medium, high) as independent factors and finisher as random intercept was performed. Additional models with an interaction between age groups and one weather variable each were performed.

**Results:** Temperature was positively associated with race time while wind speed and humidity were negatively associated (*p* < 0.001). Men were significantly greater affected wind speed and humidity than women (*p* < 0.001 for interaction) but not by temperature (*p* = 0.17 for interaction). With an average of 8 min longer race time, high temperature had the greatest effect on race time. The effect of high humidity on race time was significantly increased in 40–59 years old men and 25–65 years old women. High temperatures had an increased effect on race time in 30–64 years old men and 40–64 years old women. The inverse association between race time and high wind speed was pronounced in finishers with younger age.

**Conclusion:** Performance was lower on days with high temperature, low humidity and low wind speed. Men seemed to benefit more from higher humidity and wind speed than women. Aged (70 +) finishers were not greater affected by high temperatures.

## Introduction

Marathon running is of high popularity with increasing numbers of participants especially for master (i.e., age group) marathoners (Jokl et al., 2004) and female runners (Vitti et al., 2020). It is well known that weather conditions are likely to deteriorate and negatively impact marathon race performance (Martin, 2007). Different environmental factors such as temperature (Cheuvront and Haymes, 2001; Ely et al., 2007; Montain et al., 2007; Vihma, 2010; Knechtle et al., 2019; Nikolaidis et al., 2019b; Gasparetto and Nesseler, 2020), wind (Vihma, 2010; Knechtle et al., 2019; Nikolaidis et al., 2019b), rain (Knechtle et al., 2019; Nikolaidis et al., 2019b), and humidity (Vihma, 2010; Knechtle et al., 2019; Nikolaidis et al., 2019b) are reported to have an influence on marathon running performance.

Especially environmental temperatures seemed to have a high impact on marathon running performance (Ely et al., 2007, 2008; Vihma, 2010; El Helou et al., 2012; Knechtle et al., 2019; Gasparetto and Nesseler, 2020) where increasing air temperatures seemed to have the highest impact on marathon race times (Cheuvront and Haymes, 2001; Ely et al., 2007; Montain et al., 2007; Vihma, 2010; Gasparetto and Nesseler, 2020).

The optimal ambient temperatures for maximal running speed seemed to depend on the performance level of a marathon runner (El Helou et al., 2012). Regarding the influence of environmental temperature, differences were reported regarding the effect on performance level (Ely et al., 2007, 2008; Vihma, 2010; Gasparetto and Nesseler, 2020) where performance seems to be impaired in both faster (Ely et al., 2008; Gasparetto and Nesseler, 2020) and in slower marathon runners (Ely et al., 2007; Montain et al., 2007; Vihma, 2010). Analyses from the “Boston Marathon” showed, however, that all performance levels of marathoners were impaired with increasing temperatures (Knechtle et al., 2019). Ambient temperatures seemed also to affect the performance regarding the sex of the marathoners (Vihma, 2010). Effects of warm weather seemed to be less evident for female than male marathoners (Vihma, 2010).

Increased ambient temperatures generally reduced athletic performance (El Helou et al., 2012; Lindemann et al., 2017; Reeve et al., 2019). High ambient temperatures can lead to exertional heat illness and even to exertional heat stroke in runners (DeMartini et al., 2014). Elderly athletes (Kenny et al., 2017) and elderly active people (Kenny et al., 2015; Stapleton et al., 2015) seemed to be more affected by higher ambient temperatures. The effects of environmental conditions such as high temperatures are well-known for different groups of marathoners (i.e., female runners, male runners, elite runners, slower runners), but have not been investigated in age group (i.e., master) marathoners although their number continuously increases in large city marathons such as the “New York City Marathon” (Jokl et al., 2004; Lepers and Cattagni, 2012).

The aim of the present study was, therefore, to investigate the effect of ambient temperature on marathon running performance in master marathoners (i.e., age group runners) competing in the largest city marathon in the world, the “New York City Marathon” since its first edition in 1970. We hypothesized that performance would decrease with increasing ambient temperature, especially with increasing age of both female and male master marathoners.

## Materials and Methods

### Ethical Approval

This study was approved by the Institutional Review Board of Kanton St. Gallen, Switzerland, with a waiver of the requirement for informed consent of the participant as the study involved the analysis of publicly available data (EKSG 01-06-2010).

### The Race

The ‘‘New York City Marathon’’ is the world’s largest annual marathon with actually over 50,000 annual finishers^1^. The “New York City Marathon” first took place in 1970. Until 1975, the marathon was held in Central Park, where four laps were completed. In the first few years it was held in mid-September, from 1976 to 1985 it took place at the end of October. It has had its current date since 1986, with the exception of 1993 and 1995, when it only took place on the second Sunday in November. The race always takes place on the first Sunday in November in New York City. There was no race in 2012 due to the aftermath of Hurricane Sandy and in 2020 the race was canceled due to safety concerns resulting from the COVID-19 pandemic.

In the first marathon on September 13, 1970, 127 participants took part, of which only 55 made it to the finish. The low proportion of finishers persisted for a few years. Only since 1979 have 90% and more of the registered runners regularly crossed the finish line. The number of participants continued to increase slowly. In 1971, there were 245 runners at the start, in 1974 more than 500. This caused increasing organizational problems, as the larger the number of participants it became more difficult to count the laps for each runner. In 1976, the “New York City Marathon” was expanded to all five New York boroughs for the first time to mark the 200th anniversary of the independence of the United States.

The “New York City Marathon” is not a circuit, but goes from Fort Wadsworth on Staten Island via Brooklyn, Queens and the Bronx to Manhattan, where the finish line is in Central Park. Due to the large number of participants, the start is now in four waves (from 2008 to 2011 in three waves) with an interval of about 30 min. In each wave there are three start lanes, which are only finally united at mile 8 (12.9 kilometers). The professional runners start separately some time before the main waves, as do the participants in the wheelchair class. The professional runners start at the head of the first wave.

### Data

The athlete data was downloaded from the official New York Road Runners website^2^ using a web browser and a JavaScript code. Every athlete’s sex, age, country of origin and final race time were thus obtained. Athletes were grouped in 5-year age groups. The weather data was obtained from https://wunderground.com. The LaGuardia Airport Station was chosen as the most appropriate weather station because of its central location along the route of the “New York City Marathon” and its complete historic dataset. For each race day, we retrieved the on-site weather conditions at 1pm, a time at which most athletes would have been around the halfway point of their run.

### Statistical Analysis

The Shapiro-Wilk and Levene’s tests were applied for normality and homogeneity, respectively. The average temperature, relative humidity, and wind speed in each race day of each year were transformed into categorical variables in tertiles for low, medium, and high. Temperature: low (5.0 – 11.7°C), medium (11.8 – 17.0°C), high (17.1 – 24.5°C). Relative humidity: low (26 – 43%), medium (43.1 – 56.9%), high (57 – 100%). Wind speed: low (8.1 – 16.1 km/h), medium (16.2 – 22.5 km/h), high (22.6 – 48.3 km/h) were transformed into categorical variables in tertiles for low, medium, and high. A linear mixed model with race time (min) as dependent variable and 5-year age groups, sex, temperature, wind and relative humidity tertiles as independent factors and finisher as random intercept was performed. The mixed model was performed once including interactions between sex and weather categories and once for both sexes separately. Additionally, models for both sexes separately including an interaction between age groups and one weather variable each were performed to explore the weather effect across age groups. Further, linear mixed models were applied for a subset of top ten male and female finishers of each race. Diagnostic plots were used to assess model assumptions. The significance level was set as *p* < 0.05. All statistics were performed with R (Version 3.5.1, R Core Team, 2017).

## Results

**FIGURE 1:**
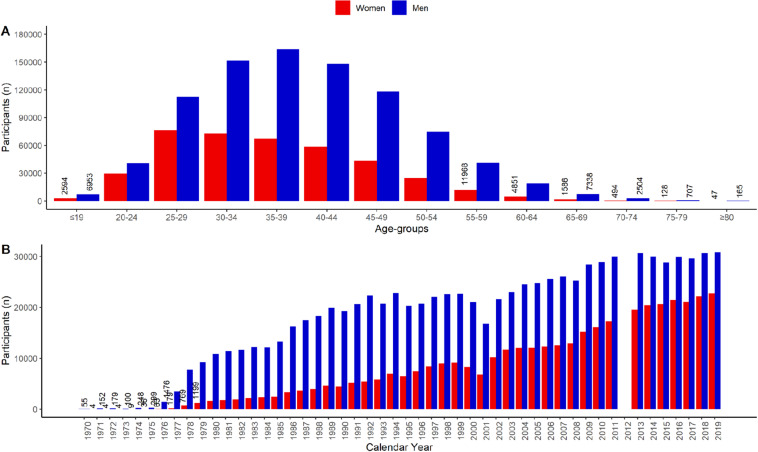
Participants by age group **(A)** and calendar years **(B)**.

**FIGURE 2:**
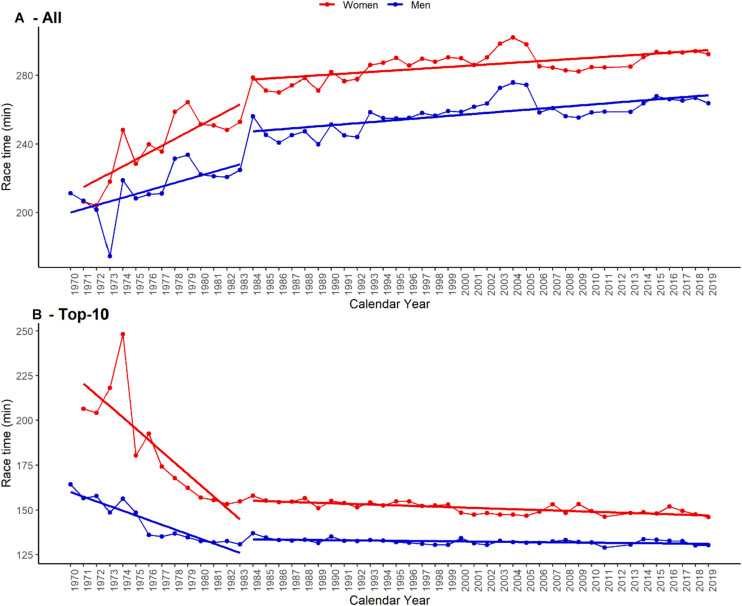
Race time across years for all finishers **(A)** and top-ten finishers **(B)**.

This analysis included data from the “New York City Marathon” from the years 1970 to 2019, including 886,569 male participants, and 393,988 female participants. Thus, the total sample size is *n* = 1,280,557. Participants aging from 30 to 49 years old were the most prevalent for both men and women (Figure 1, panel A). The number of participants is increasing for both sexes across years (Figure 1, panel B). Although the number of women is increasing more than men throughout the years, women only represented 42% of participants in 2019. Along with the increasing number of participants, race time has increased (Figure 2, panel A). On the other hand, the top ten finishers improved their race time within the first decade (Figure 2, panel B).

**FIGURE 3:**
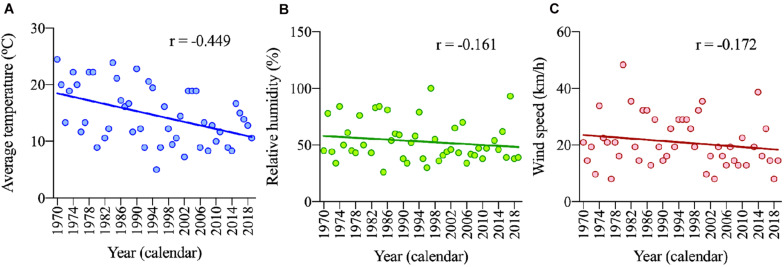
Change in temperature **(A)**, humidity **(B)** and wind speed **(C)** across years.

Table 1 shows the weather conditions (temperature, humidity, and wind speed) in the “New York City Marathon” on race day with data from 1970 to 2019. A negative correlation was identified between average temperature and calendar year (*p* = 0.001), but not between calendar year and humidity or wind speed (Figure 3).

**TABLE 1:** Weather conditions in the “New York City Marathon” on race day.

	**Mean**	**SD**	**Min**	**Max**
Average temperature (∘C)	14.6	5.1	5.0	24.4
Relative humidity (%)	53.1	17.9	26.0	100.0
Wind speed (km/h)	21.0	8.9	8.0	48.3

*Data from 1970 to 2019. SD, standard deviation.*

**FIGURE 4:**
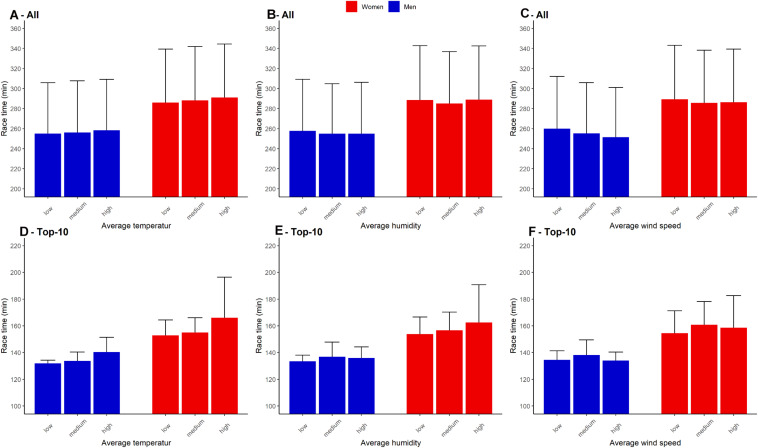
Race time by performance level for temperature **(A)**, humidity **(B)** and wind speed **(C)** for all finishers and for top ten finishers **(D–F)**.

**FIGURE 5:**
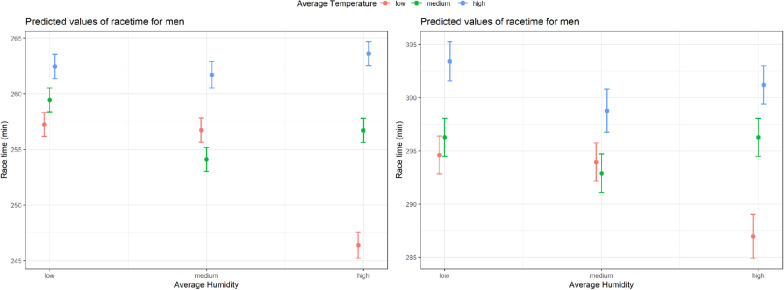
Predicted average race time (min) with 95% confidence intervals for men and women according to temperature and humidity, derived from the linear mixed models.

Mean (SD) race time by weather categories (temperature, humidity, and wind speed) by sex is shown for all finishers and top ten finishes in Figure 4. We found significant interactions for sex and wind speed (*p* < 0.001) as well as sex and humidity (*p* < 0.001) within the whole study cohort, but not within top ten finishers. Thereof, Table 2 shows the linear mixed models for both sexes separately for the full cohort and the model including both, men and women, for top ten finishers. The greatest effect size was found for high temperatures in men [β 7.73 95% Confidence Interval (7.5 – 7.97)] and women [β 7.78 (7.37 – 8.29)] as well as top ten finishers [β 1.87 (0.78 – 2.97)]. For a sensitivity analyses, we included an interaction term between humidity and temperature. In men, high temperature in combination with high humidity were associated with highest race time while low temperature and high humidity were related to lowest race time. In women, highest race time was observed in high temperature and low humidity (Figure 5).

**TABLE 2:** Linear mixed model with random intercept for race time (min).

	**Model 1 (Full cohort men)**	**Model 2 (Full cohort women)**	**Model 3 (top-10)**
Medium humidity	−1.73 [−1.92; −1.54] *	−1.74 [−2.04; −1.45] *	0.08 [−0.94; 1.10]
High humidity	−2.11 [−2.31; −1.90] *	−0.90 [−1.24; −0.57] *	0.56 [−0.40; 1.52]
Medium temperature	1.19 [1.00; 1.38] *	1.38 [1.09; 1.67] *	0.04 [−0.92; 0.99]
High temperature	7.73 [7.50; 7.97] *	7.78 [7.37; 8.19] *	1.87 [0.78; 2.97] *
Medium wind speed	−3.18 [−3.39; −2.98] *	−1.90 [−2.22; −1.57] *	1.51 [0.55; 2.48] *
High wind speed	−4.92 [−5.12; −4.73] *	−0.87 [−1.18; −0.57] *	1.35 [0.35; 2.36] *
Male sex	N/A	N/A	−24.24 [−26.7; −21.8] *
Num. obs.	886471	393985	949
Num. groups: id	620308	301502	661
Var: id (Intercept)	1693.04	2044.45	236.85
Var: Residual	683.23	614.62	11.94

Shown are β effect size with 95% confidence intervals. *p < 0.05, low tertile for age categories (not shown) and each weather variable. All models were adjusted for age categories (not shown).

**FIGURE 6:**
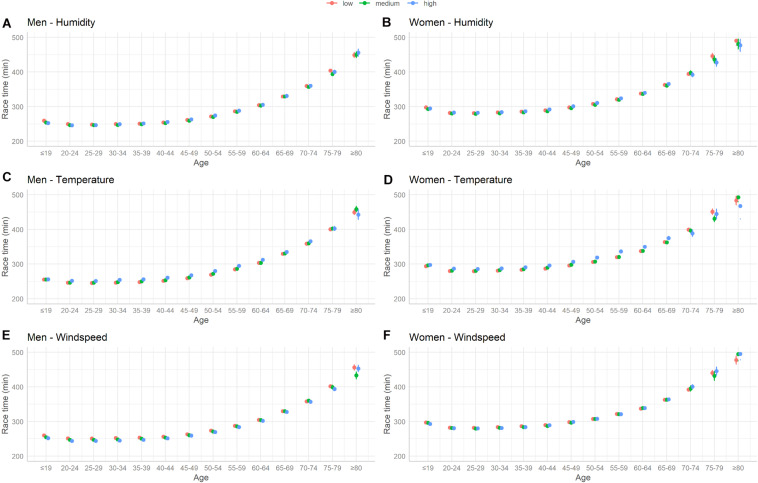
Predicted average race time (min) with 95% confidence intervals for each age group and weather category derived from the linear mixed models. **(A)** Men–humidity. **(B)** Women–humidity. **(C)** Men–temperature. **(D)** Women–temperature. **(E)** Men–wind speed. **(F)** Women–wind speed.

We observed a non-linear relation between race time and age with fastest race time in age 20–34 and increasing race time afterward. We found a significant interaction between age groups and each weather variable. The relation between age group, weather condition and race time is depicted in Figure 6, showing the predicted average race time and 95% confidence intervals for each age group and weather category, derived from the linear mixed models.

The effect of high humidity on race time was increased in age groups 40–59 years in men and 25–65 years in women (Table 3). High temperatures had an increased effect on race time in age groups 30–64 years in men and 40–64 years in women (Table 4). The inverse association between race time and high wind speed was pronounced in finishers with younger age and less strong in finishers in age groups 40 + (Table 5).

**TABLE 3:** Linear mixed model for race time with age group and humidity interaction.

	**Men**	**Women**
(Intercept)	298.38 [297.73; 299.02]*	334.14 [332.86; 335.42]*
Age group [S. = 19]	−39.02 [−40.64; −37.40]*	−36.38 [−39.10; −33.65]*
Age group [S.20–24]	−48.75 [−49.62; −47.87]*	−52.29 [−53.74; −50.83]*
Age group [S.25–29]	−50.09 [−50.82; −49.35]*	−53.08 [−54.42; −51.73]*
Age group [S.30–34]	−48.82 [−49.53; −48.11]*	−50.65 [−52.00; −49.30]*
Age group [S.35–39]	−47.41 [−48.11; −46.71]*	−48.65 [−50.00; −47.30]*
Age group [S.40–44]	−44.28 [−44.98; −43.58]*	−45.07 [−46.43; −43.71]*
Age group [S.45–49]	−36.96 [−37.68; −36.25]*	−36.11 [−37.49; −34.74]*
Age group [S.50–54]	−26.51 [−27.26; −25.77]*	−26.06 [−27.50; −24.62]*
Age group [S.55–59]	−12.03 [−12.83; −11.23]*	−12.63 [−14.20; −11.07]*
Age group [S.60–64]	5.96 [5.02; 6.90]*	3.64 [1.80; 5.47]*
Age group [S.65–69]	31.07 [29.84; 32.31]*	28.82 [26.22; 31.43]*
Age group [S.70–74]	61.02 [59.17; 62.87]*	60.36 [56.29; 64.42]*
Age group [S.75–79]	105.37 [102.09; 108.66]*	112.08 [105.11; 119.04]*
Medium Humidity	−2.42 [−3.31; −1.52]*	−3.02 [−4.73; −1.32]*
Age group [S. = 19]	−2.82 [−5.23; −0.40]*	−1.87 [−6.03; 2.29]
Age group [S.20–24]	−0.73 [−2.01; 0.55]	1.24 [−0.78; 3.25]
Age group [S.25–29]	0.60 [−0.44; 1.63]	0.87 [−0.95; 2.69]
Age group [S.30–34]	0.17 [−0.83; 1.16]	0.47 [−1.35; 2.29]
Age group [S.35–39]	0.68 [−0.31; 1.67]	1.13 [−0.70; 2.96]
Age group [S.40–44]	0.88 [−0.11; 1.87]	0.60 [−1.25; 2.44]
Age group [S.45–49]	0.62 [−0.40; 1.63]	0.42 [−1.47; 2.32]
Age group [S.50–54]	0.57 [−0.50; 1.64]	0.14 [−1.87; 2.15]
Age group [S.55–59]	0.84 [−0.35; 2.02]	1.06 [−1.20; 3.32]
Age group [S.60–64]	1.08 [−0.33; 2.48]	2.10 [−0.72; 4.92]
Age group [S.65–69]	2.02 [0.11; 3.94]*	1.04 [−2.99; 5.07]
Age group [S.70–74]	0.06 [−2.85; 2.97]	6.24 [−0.13;12.62]
Age group [S.75–79]	−7.45 [−12.51; −2.38]*	−7.25 [−18.69; 4.19]
High Humidity	0.09 [−0.88; 1.07]	−1.32 [−3.19; 0.55]
Age group [S. = 19]	−7.25 [−9.60; −4.91]*	−2.05 [−6.12; 2.02]
Age group [S.20–24]	−3.46 [−4.76; −2.16]*	2.09 [−0.05; 4.23]
Age group [S.25–29]	−1.57 [−2.67; −0.47]*	2.69 [0.72; 4.67]*
Age group [S.30–34]	−0.64 [−1.71; 0.42]	2.00 [0.02; 3.97]*
Age group [S.35–39]	0.19 [−0.87; 1.25]	2.66 [0.67; 4.64]*
Age group [S.40–44]	1.35 [0.29; 2.42]*	4.17 [2.16; 6.17]*
Age group [S.45–49]	1.67 [0.58; 2.76]*	4.23 [2.18; 6.28]*
Age group [S.50–54]	2.14 [1.00; 3.29]*	3.64 [1.47; 5.81]*
Age group [S.55–59]	1.88 [0.63; 3.14]*	3.99 [1.57; 6.41]*
Age group [S.60–64]	1.02 [−0.50; 2.54]	3.37 [0.41; 6.32]*
Age group [S.65–69]	1.11 [−0.93; 3.16]	3.73 [−0.45; 7.91]
Age group [S.70–74]	0.71 [−2.28; 3.70]	−0.80 [−7.56; 5.96]
Age group [S.75–79]	−3.77 [−9.10; 1.56]	−17.66 [−29.42; −5.90]*
Num. obs.	886471	393985
Num. groups: id	620308	301502
Var: id (Intercept)	1691.19	2041.32
Var: Residual	697.24	621.97

Shown are β effect size with 95% Confidence Intervals.* Null hypothesis value outside the 95% confidence interval. Grand Mean as reference for age groups and Low category as reference for humidity.

**TABLE 4:** Linear mixed model for race time with age group and temperature interaction.

	**Men**	**Women**
(Intercept)	296.21 [295.56; 296.86]*	332.90 [331.64; 334.15]*
Age group [S. = 19]	−41.01 [−42.65; −39.37]*	−39.26 [−42.00; −36.53]*
Age group [S.20–24]	−50.00 [−50.89; −49.11]*	−52.97 [−54.41; −51.53]*
Age group [S.25–29]	−50.77 [−51.50; −50.03]*	−53.63 [−54.96; −52.31]*
Age group [S.30–34]	−49.96 [−50.68; −49.25]*	−51.71 [−53.03; −50.38]*
Age group [S.35–39]	−48.17 [−48.88; −47.47]*	−49.53 [−50.85; −48.20]*
Age group [S.40–44]	−44.73 [−45.43; −44.02]*	−46.20 [−47.53; −44.87]*
Age group [S.45–49]	−37.20 [−37.92; −36.49]*	−37.02 [−38.37; −35.67]*
Age group [S.50–54]	−26.74 [−27.49; −26.00]*	−26.87 [−28.28; −25.45]*
Age group [S.55–59]	−11.66 [−12.46; −10.85]*	−12.78 [−14.32; −11.24]*
Age group [S.60–64]	7.23 [6.29; 8.17]*	4.51 [2.66; 6.36]*
Age group [S.65–69]	33.15 [31.91; 34.40]*	30.50 [27.90; 33.11]*
Age group [S.70–74]	62.59 [60.72; 64.46]*	66.26 [62.17; 70.35]*
Age group [S.75–79]	104.02 [100.68; 107.37]*	118.10 [110.71; 125.49]*
Medium Temperature	1.92 [1.10; 2.75]*	−0.00 [−1.53; 1.53]
Age group [S. = 19]	−1.87 [−4.17; 0.43]	2.65 [−1.15; 6.45]
Age group [S.20–24]	−1.82 [−3.00; −0.64]*	0.82 [−0.99; 2.64]
Age group [S.25–29]	−0.87 [−1.83; 0.09]	1.03 [−0.61; 2.67]
Age group [S.30–34]	−0.41 [−1.33; 0.51]	1.44 [−0.20; 3.08]
Age group [S.35–39]	−0.03 [−0.94; 0.88]	2.01 [0.37; 3.66]*
Age group [S.40–44]	−0.04 [−0.95; 0.87]	2.64 [0.98; 4.30]*
Age group [S.45–49]	0.34 [−0.59; 1.28]	2.06 [0.36; 3.76]*
Age group [S.50–54]	0.24 [−0.75; 1.22]	1.22 [−0.58; 3.02]
Age group [S.55–59]	−0.32 [−1.41; 0.77]	0.23 [−1.78; 2.24]
Age group [S.60–64]	−1.83 [−3.14; −0.53]*	0.51 [−1.97; 2.99]
Age group [S.65–69]	−0.51 [−2.26; 1.24]	−0.94 [−4.51; 2.63]
Age group [S.70–74]	−0.85 [−3.47; 1.76]	−2.68 [−8.25; 2.88]
Age group [S.75–79]	0.60 [−4.10; 5.30]	−20.33 [−30.55; −10.11]*
High temperature	5.99 [4.76; 7.22]*	5.00 [1.94; 8.06]*
Age group [S. = 19]	−5.14 [−7.70; −2.58]*	−1.35 [−6.43; 3.72]
Age group [S.20–24]	−0.72 [−2.26; 0.82]	1.68 [−1.62; 4.97]
Age group [S.25–29]	−0.12 [−1.46; 1.23]	1.34 [−1.81; 4.50]
Age group [S.30–34]	1.82 [0.51; 3.13]*	1.34 [−1.81; 4.49]
Age group [S.35–39]	2.09 [0.79; 3.40]*	1.93 [−1.23; 5.08]
Age group [S.40–44]	3.06 [1.74; 4.37]*	3.96 [0.78; 7.14]*
Age group [S.45–49]	2.89 [1.55; 4.23]*	5.50 [2.26; 8.74]*
Age group [S.50–54]	4.58 [3.18; 5.98]*	7.74 [4.35; 11.12]*
Age group [S.55–59]	4.18 [2.65; 5.71]*	11.27 [7.61; 14.94]*
Age group [S.60–64]	3.08 [1.28; 4.89]*	7.62 [3.24; 12.00]*
Age group [S.65–69]	−0.66 [−3.12; 1.81]	6.66 [0.96; 12.36]*
Age group [S.70–74]	1.17 [−2.47; 4.81]	−15.36 [−25.00; −5.72]*
Age group [S.75–79]	−3.57 [−9.74; 2.59]	−11.48 [−26.09; 3.12]
Num. obs.	886471	393985
Num. groups: id	620308	301502
Var: id (Intercept)	1703.97	2044.41
Var: Residual	683.04	614.85

Shown are β effect size with 95% confidence intervals.* Null hypothesis value outside the 95% confidence interval. Grand Mean as reference for age groups and Low category as reference for temperature.

**TABLE 5:** Linear mixed model for race time with age group and wind speed interaction.

	**Men**	**Women**
(Intercept)	299.88 [299.22; 300.53]*	332.77 [331.60; 333.93]*
Age group [S. = 19]	−40.16 [−41.79; −38.53]*	−35.46 [−38.12; −32.80]*
Age group [S.20–24]	−48.37 [−49.26; −47.49]*	−50.38 [−51.73; −49.03]*
Age group [S.25–29]	−49.13 [−49.88; −48.39]*	−50.98 [−52.22; −49.74]*
Age group [S.30–34]	−47.69 [−48.41; −46.97]*	−48.65 [−49.89; −47.41]*
Age group [S.35–39]	−46.35 [−47.06; −45.64]*	−46.11 [−47.34; −44.87]*
Age group [S.40–44]	−43.81 [−44.53; −43.10]*	−43.09 [−44.34; −41.85]*
Age group [S.45–49]	−36.91 [−37.63; −36.18]*	−34.70 [−35.96; −33.44]*
Age group [S.50–54]	−26.40 [−27.15; −25.65]*	−25.22 [−26.54; −23.90]*
Age group [S.55–59]	−12.29 [−13.09; −11.48]*	−11.12 [−12.56; −9.69]*
Age group [S.60–64]	4.62 [3.68; 5.55]*	4.65 [2.95; 6.36]*
Age group [S.65–69]	29.93 [28.71; 31.15]*	29.90 [27.52; 32.28]*
Age group [S.70–74]	58.37 [56.59; 60.14]*	59.36 [55.56; 63.17]*
Age group [S.75–79]	102.01 [98.96; 105.06]*	107.13 [100.74; 113.52]*
Medium Wind Speed	−3.89 [−4.90; −2.88]*	−0.03 [−2.01; 1.95]
Age group [S. = 19]	−1.21 [−3.78; 1.36]	−1.34 [−5.82; 3.14]
Age group [S.20–24]	−0.58 [−1.97; 0.82]	−1.18 [−3.47; 1.11]
Age group [S.25–29]	0.14 [−1.02; 1.29]	−1.93 [−4.02; 0.17]
Age group [S.30–34]	−0.08 [−1.19; 1.03]	−2.13 [−4.22; −0.03]*
Age group [S.35–39]	0.52 [−0.58; 1.62]	−2.52 [−4.62; −0.41]*
Age group [S.40–44]	1.38 [0.28; 2.49]*	−2.35 [−4.47; −0.24]*
Age group [S.45–49]	1.27 [0.14; 2.41]*	−1.51 [−3.68; 0.66]
Age group [S.50–54]	1.41 [0.22; 2.60]*	0.07 [−2.23; 2.36]
Age group [S.55–59]	2.39 [1.08; 3.70]*	−0.30 [−2.86; 2.26]
Age group [S.60–64]	3.60 [2.04; 5.15]*	1.18 [−1.97; 4.33]
Age group [S.65–69]	3.56 [1.46; 5.67]*	0.26 [−4.21; 4.73]
Age group [S.70–74]	5.65 [2.49; 8.80]*	2.95 [−4.83; 10.73]
Age group [S.75–79]	1.15 [−4.48; 6.79]	−8.54 [−21.25; 4.18]
High Wind Speed	−5.00 [−5.96; −4.04]*	1.45 [−0.41; 3.30]
Age group [S. = 19]	−2.84 [−5.13; −0.56]*	−6.05 [−10.04; −2.07]*
Age group [S.20–24]	−2.64 [−3.92; −1.36]*	−3.31 [−5.43; −1.19]*
Age group [S.25–29]	−1.93 [−3.01; −0.85]*	−3.34 [−5.30; −1.38]*
Age group [S.30–34]	−2.37 [−3.41; −1.32]*	−4.10 [−6.06; −2.14]*
Age group [S.35–39]	−1.62 [−2.66; −0.58]*	−4.49 [−6.46; −2.52]*
Age group [S.40–44]	0.18 [−0.86; 1.23]	−2.08 [−4.07; −0.10]*
Age group [S.45–49]	1.33 [0.26; 2.40]*	−0.73 [−2.76; 1.30]
Age group [S.50–54]	0.88 [−0.24; 2.00]	−0.92 [−3.06; 1.22]
Age group [S.55–59]	1.04 [−0.19; 2.27]	−1.66 [−4.05; 0.74]
Age group [S.60–64]	2.72 [1.25; 4.19]*	−0.07 [−3.00; 2.86]
Age group [S.65–69]	2.81 [0.84; 4.77]*	−0.36 [−4.52; 3.79]
Age group [S.70–74]	3.70 [0.79; 6.61]*	6.96 [0.63; 13.29]*
Age group [S.75–79]	−3.15 [−8.33; 2.02]	3.87 [−8.49; 16.23]
Num. obs.	886471	393985
Num. groups: id	620308	301502
Var: id (Intercept)	1683.29	2041.29
Var: Residual	695.84	623.37

Shown are β effect size with 95% confidence intervals.* Null hypothesis value outside the 95% confidence interval. Grand Mean as reference for age groups and Low category as reference for wind speed.

## Discussion

This study investigated the effect of environmental conditions (i.e., ambient temperature, humidity, precipitation, and wind speed) on marathon running performance in age group marathoners competing in the largest city marathon in the world, the “New York City Marathon” with the hypothesis that performance would decrease with increasing ambient temperature, especially with increasing age of both female and male age group marathoners. The main findings were (i) temperature was positively associated with race time while wind speed and humidity were negatively associated, (ii) men were significantly greater affected by wind speed and humidity than women but not by temperature, (iii) the effects were smaller and did not differ between men and women in the top ten finishers, (iv) the effect of high humidity on race time was increased in age groups 40–59 years in men and 25–65 years in women, (v) high temperatures had an increased effect on race time in age groups 30–64 years in men and 40–64 years in women, and (vi) the inverse association between race time and high wind speed was pronounced in finishers with younger age and less strong in finishers in age groups 40 +.

We can confirm our hypothesis that increasing ambient temperatures is associated with reduced marathon race time regardless of sex. This association was found in all finishers as well as in top-10 finishers only. The effect of high temperature on race time was significantly greater in 30–65 years old men and in 40–69 years old women, but not greater in the very old (70 +) in contrast to our hypotheses. The influence of ambient temperature on marathon running performance is well-known (Ely et al., 2007, 2008; Vihma, 2010; Knechtle et al., 2019; Gasparetto and Nesseler, 2020), however, increased temperatures seemed to have different effects on different groups of runners (e.g., slower and faster runners, younger and older runners, female and male runners).

In a large city marathon such as the “New York City Marathon,” the fastest race times are achieved by runners at younger ages (Nikolaidis et al., 2018b). In the present analysis, the plateau of the fastest race times ranged for both men and women from 30 to 40 years old, regardless of the decade. However, most of the runners in the “New York City Marathon” are master runners (Jokl et al., 2004) who are considerably slower than elite runners (Nikolaidis and Knechtle, 2019). Therefore, one might assume that age group marathoners would suffer more from increased ambient temperatures due to their slower race times.

Generally, slower marathoners seemed to be more affected by increased temperatures (Ely et al., 2007; Vihma, 2010). However, other studies reported that increasing temperatures slowed also faster marathoners (Ely et al., 2008). Regarding the “New York City Marathon,” Gasparetto and Nesseler (2020) investigated performances of the top 1,000 runners for every year during the last twelve editions and found that the fastest runners experienced a larger decline in performance than the slower ones under identical thermal exposures. These disparate findings might be explained by the different approaches to the analyses (e.g., selection of elite and sub-elite runners, definition of high and low temperatures, sample sizes, etc.).

We also found differences regarding the sexes. A potential explanation for the disparate findings for female and male age group runners could be the lower female participation in this race (42% female finishers in 2019). Little is known in literature regarding a potential sex difference of the influence of ambient temperature in marathon running. One study found a difference in the effect of warm weather for female and male marathoners. An analysis of different weather variables on running performance in the “Stockholm Marathon” from 1980 to 2008 showed that effects of warm weather were less evident for female than for male runners (Vihma, 2010). Future studies need to investigate the sex differences regarding the influence of temperature on female and male marathon running performance.

One might also assume that a decrease in marathon running performance due to increased ambient temperatures might be due to global warming (El Helou et al., 2012). In the “New York City Marathon,” however, temperatures on race day decreased across calendar years. This might be due to the fact that race date changed from September to October and later to November.

Regarding the other weather variables, we found that wind speed and humidity were negatively associated with race time (i.e., faster race times with higher wind speed and higher humidity). Men were significantly greater affected by medium and high wind speed and high humidity than women and the effects were smaller and did not differ between men and women in the top ten finishers. For the age group runners, the effect of high humidity on race time was increased in age groups 40–59 years in men and 25–65 years in women, and the inverse association between race time and high wind speed was pronounced in finishers with younger age and less strong in finishers in age groups 40 +.

The aspect of the influence of wind has been investigated in the “Boston Marathon” as a point-to-point race. In the “Boston Marathon,” wind affected marathon running performance (El Helou et al., 2012; Knechtle et al., 2019). Tail wind improved performances (Knechtle et al., 2019) but increasing wind speed was also related to worsened performances in all finishers and near elite groups (Knechtle et al., 2019). Wind coming from the West, compared to wind coming from other directions, was the most favorable for performance (Knechtle et al., 2019). The difference between the “New York City Marathon” and the “Boston Marathon” regarding the influence of wind on marathon running performance are very likely explained by their setting. The “New York City Marathon” is held in a city and not a point-to-point race in contrast to the “Boston Marathon.” As far as we are aware, no other study found differences regarding the influence of wind speed in marathon running between the sexes. Future studies need to investigate why men seemed to benefit more from higher humidity and wind speed than women with regard to their race time.

While we found an influence of humidity on marathon running performance, others found not. Although one might assume that humidity might affect marathon running performance, an analysis of the influence of temperature, humidity, dew point, and the atmospheric pressure at sea level in six European (Paris, London, Berlin) and American (Boston, Chicago, New York) marathon races from 2001 to 2010 through 1,791,972 participants’ performances (all finishers per year and race) showed that air temperature, but no other environmental parameters had any significant impact on marathon running performance (El Helou et al., 2012). The disparate findings might be explained by the fact that we investigated one single race held always at the same location where El Helou et al. (2012) combined data of different race locations. Future studies need to investigate the differences between female and male marathon runners regarding the effect of wind speed and humidity and their performance.

Specific physiological mechanisms might, however, also explain sex- and age-dependent influences on marathon running performance in master marathoners where age, sex, anthropometry, fitness level and training might have an influence on heat tolerance (Kazman et al., 2015; Alele et al., 2021). Heat tolerance can be influenced by heat acclimation leading to a reduction in heat stress (Garrett et al., 2014; Schleh et al., 2018). The fitness level has an influence on heat tolerance, where low cardiorespiratory fitness (Selkirk and McLellan, 2001; Lisman et al., 2014) and body fatness (Selkirk and McLellan, 2001) are associated with heat intolerance. Individuals with a higher body fat have a lower heat tolerance due to a reduced capacity to store heat (Cheung et al., 2000). Training can improve heat tolerance (McLellan, 2001) where the training must be long enough to train heat tolerance (Cheung and McLellan, 1999). Women are more likely to be heat intolerant than men (Druyan et al., 2012; Kazman et al., 2015). Thermoregulation in women is affected by the menstrual cycle where body core temperature is adversely affected during the luteal phase (Pivarnik et al., 1992). Women not using oral contraceptives are at a thermoregulatory disadvantage during the luteal phase of the menstrual cycle (Cheung et al., 2000). Heat tolerance is increased during early follicular phase for non-users of oral contraceptives (Tenaglia et al., 1999).

### Limitations

A limitation is the aspect that the first races were held in “Central Park” with trees and the race then was held as a city marathon with a difference course. The shadow from the trees might have had an influence on performance. Other aspects such as psychological and physiological aspects (Nikolaidis and Knechtle, 2018; Nikolaidis et al., 2018a), pre-race experience (Malchrowicz-Mośko et al., 2020), training (Piacentini et al., 2013), and nutrition (Roca et al., 2020; Knechtle et al., 2021; Methenitis et al., 2021) and pacing during the race (Cuk et al., 2019; Nikolaidis et al., 2019a) were not considered.

## Conclusion

In 1,280,557 age group finishers the “New York City Marathon” from the years 1970 to 2019, temperature was positively associated with race time while wind speed and humidity were negatively associated. Regarding sex, men were significantly greater affected by wind speed and humidity than women but not by temperature. High temperatures had the greatest effect on race time with an average of 8 min longer race time. Regarding age, the effect of high humidity on race time was significantly increased in 40–59 years old men and 25–65 years old women. High temperatures had an increased effect on race time in 30–64 years old men and 40–64 years old women. The inverse association between race time and high wind speed was pronounced in finishers with younger age. An observational study investigating a large data set provides results from “real life” enabling athletes and coaches to better prepare for “real life conditions.”

## Data Availability Statement

The raw data supporting the conclusions of this article will be made available by the authors, without undue reservation.

## Author Contributions

EV, PN, CM, OG, CS, and BK performed the material preparation and data collection. TM and CS performed the data analysis. BK, TM, and CS conducted the data interpretation. BK wrote the first draft of the manuscript. All authors contributed to the study conception and design, commented on previous versions of the manuscript, read, and approved the final manuscript.

## Conflict of Interest

The authors declare that the research was conducted in the absence of any commercial or financial relationships that could be construed as a potential conflict of interest.

## Footnotes

1 https://www.nyrr.org/tcsnycmarathon

2 https://results.nyrr.org
